# Association Between American Society of Anesthesiologists Physical Status Classification System and Remimazolam Sedation Outcomes

**DOI:** 10.1111/acem.70306

**Published:** 2026-04-27

**Authors:** Caroline H. Brynning, Simone P. Busch, Anne L. Krarup, Dorte Melgaard

**Affiliations:** ^1^ Department of Emergency Medicine and Trauma Center, EMRUn Aalborg University Hospital Aalborg Denmark; ^2^ Department of Clinical Medicine Aalborg University Aalborg Denmark

## Abstract

**Background:**

Remimazolam (RM) is an ultrashort‐acting benzodiazepine with pharmacodynamic properties ideal for procedural sedation in emergency departments (EDs). This study evaluated the safety of RM when used for procedural sedation of American Society of Anesthesiologists (ASA) I–III patients in the ED.

**Aims:**

To compare the occurrence of no or mild/moderate respiratory complications during procedural sedation in patients with ASA scores I, II, and III. Secondarily, to compare clinical characteristics of RM sedations between the ASA groups.

**Methods:**

This qualitative improvement study was conducted in 2024 and 2025 at a Danish Level 1 trauma center. RM was administered to ASA I–III patients ≥ 12 years of age as procedural sedation by emergency medicine physicians or nurses educated in airway management, RM sedation, and adverse events. Safety was measured by the occurrence of respiratory problems graded as none, mild, moderate, or severe.

**Results:**

Of the 540 patients sedated with RM, 95%–100% in ASA I–III had no or mild/moderate respiratory complications, with ASA I and III being the safest groups (*p* < 0.014). ASA III patients had the most potential airway risks, but still experienced fewer severe respiratory complications than ASA II patients (4.7% vs. 1.3%, *p* = 0.014). The RM dose was found to be age‐, gender‐ and ASA score‐dependent.

**Conclusions:**

RM showed great potential for procedural sedation and was safe for procedural sedation of ASA I–III patients in the ED, with few serious respiratory complications, even in high doses and with high‐risk profiles.

**Abbreviations**
ASA: American Society of Anesthesiologists
BMI: Body Mass Index
ED: Emergency Department
IQR: Interquartile Range
L: Liters
mg: Milligrams
min: Minutes
*n*
: Number
OR: Operating Room
RCT: Randomized clinical trial
RM: Remimazolam
μg: Micrograms

## 1 Introduction

Opioids and benzodiazepines are frequently used for sedation in emergency departments (EDs), allowing patients to undergo procedures safely and comfortably while maintaining stable cardiorespiratory function [1, 2]. Currently, the most commonly used agents for ambulatory sedation are midazolam, propofol, ketamine, dexmedetomidine, and remifentanil [3]. However, remimazolam (RM), an ester‐based ultrashort‐acting benzodiazepine, has demonstrated superior characteristics. It has a rapid onset of 1–3 min and a short half‐life of approximately 10 min; it is metabolized by serum esterases, resulting in no active metabolites, a faster recovery time, a higher procedural success rate, and earlier patient discharge compared to conventional agents. Furthermore, RM clearance is independent of renal and hepatic function [4, 5]. RM is associated with hemodynamic stability, a readily available antidote, and a lower risk of hypotension, bradycardia, respiratory depression, and injection‐related pain compared to propofol [4, 6, 7, 8]. Its pharmacological profile enables shorter procedures, potentially optimizing the availability of operating theaters and postsedation care units, ultimately improving access to healthcare and generating savings for both hospitals and healthcare systems [6]. Procedural sedation of American Society of Anesthesiologists (ASA) Physical Status Classification III/IV patients is associated with an increased risk of adverse events [8]. However, recent studies suggest that RM is both safe and effective for procedural sedation in ASA III/IV patients [9, 10]. Currently, there is limited research documenting the use of RM in the ED. However, a study by our colleagues found the use of RM for procedural sedation in the ED to be safe and effective with high patient satisfaction levels [11]. Our hypothesis was that RM could be administered safely in ASA I, II, and III patients for procedural sedation by ED nurses and doctors. This study aims to determine whether (1) there is a difference in the percentage of patients with no or mild/moderate respiratory complications according to the ASA score, and (2) the required RM dosage differs depending on ASA score, weight, age, or concomitant opioid treatment.

## 2 Methods

### 2.1 Study Design

This prospective clinical quality study was conducted between 10/5/24 and 4/9/25 at the Aalborg University Hospital ED. The department is a Level 1 trauma center and receives all types of acute injuries and illnesses, including approximately 30,000 orthopedic surgery patients annually. This study received approval and registration (ID 1017–011259) as a quality study by the Aalborg University Hospital Administration. All patients provided oral consent for sedation with RM, which was documented in their medical records. In Denmark, written consent to participate in a quality study using an already approved drug is not required, and quality studies do not require approval from the National Center for Ethics.

### 2.2 Study Population

In this study, both emergency medicine physicians and nurses, experienced in airway management and the management of adverse events, were trained in the use of RM prior to administering it for procedural sedation. Patient assignment was based on the availability of providers trained to administer RM during ED shifts.

Inclusion criteria were ASA score I–III, age ≥ 12 years, expected tolerance to benzodiazepines and opioids, and procedures expected to cause pain or anxiety. Exclusion criteria were ABCDE instability prior to the procedure, known intolerance to benzodiazepines and opioids, head trauma, severe intoxication, a diagnosis of myasthenia gravis, or if the individual ED nurse performing the sedation assessed the patient as having too many risk factors for RM sedation in the ED setting.

### 2.3 Outcome Measures

The primary outcome was measured as the percentage of patients experiencing no, mild, or moderate respiratory complications during RM sedation. In this study, the anticipated patient responses to procedural sedation and corresponding interventions were defined as respiratory complications and will be addressed as such throughout the article. Mild respiratory complications were defined as requiring one of the following interventions during sedation: talking to the patient, giving the patient's shoulder a gentle shake, or increasing oxygen supplementation. Moderate respiratory complications were defined as requiring one of the following: initiation of the painful procedure, performing a jaw thrust, or inserting a nasopharyngeal airway. Severe respiratory complications were defined as requiring manual assisted ventilation or administration of an antidote.

Secondary outcomes were defined as RM dosage, the type of opioid and dosage administered before or during sedation, with all measures compared across ASA scores I–III.

### 2.4 Data Collection

Included demographic and medical data were age, gender, height, weight, body mass index (BMI), ASA score, procedure type, risk factors for problematic airway handling, and administration of relevant drugs measured in doses of milligrams (mg) or micrograms (μg). The ASA Physical Status Classification System is a standardized method in perioperative medicine, using comorbidities and a variety of patient factors to assess the physiological status of patients before surgery and helps predict the risks associated with surgery or operative intervention [12]. Safety was measured by the occurrence of respiratory problems graded as mild, moderate, or severe, based on the required intervention. Sedation and procedural effectiveness were measured by the time from administration of the first dose to the start of the procedure, the proportion of patients sleeping at the start of the procedure, and whether patients were safe to leave alone after 20 min. Both staff and patients evaluated the sedation after the patient was cognitively recovered. Patient and provider satisfaction were measured by the proportion of patients with amnesia for the procedure, patients exhibiting signs of pain during the procedure, and providers evaluating the procedure as a success. Pain was categorized as mild (frowning, subtle noises, brief eye‐opening during the most painful part), moderate (moaning, expressing pain, tensing up, repeatedly opening the eyes), or severe (clear expression of pain, evasive maneuvers, constant eye opening). Data were collected after the procedure and recorded by the physician responsible for the sedation.

### 2.5 Study Protocol

#### 2.5.1 Preprocedural Assessments

In accordance with local guidelines [13], we assessed patients based on ASA score, BMI, neck mobility, presence of a large tongue, or history of sleep apnea. The quantity of opioids administered prehospital or preprocedure was noted and evaluated. Patients were preoxygenated with 3 L/min, and vital parameters were continuously monitored. Blood pressure was measured every fifth minute. The suction system and ventilation bag were prepared and tested prior to sedation. Opioids could be administered before sedation if high intraprocedural pain was anticipated, or in small doses if the aim was to enhance sedation.

#### 2.5.2 Intraprocedural Assessments

According to the guideline, patients were initially sedated with 2.5–5.0 mg of RM, followed by refractory doses of 2.5 mg RM until a state of clear relaxation. The initial dose was calculated based on patient biometrics, age, weight, comorbidity, opioids already administered, and preprocedural pain [14]. If sedation and analgesia were insufficient, top‐off doses of 2.5 mg RM and/or alfentanil could be administered until the desired effect was achieved. Vital signs and sedation were continuously measured. In case of complications, the ventilation bag and antidotes (naloxone and flumazenil) were readily available near the patient in the room. In case of decreased blood oxygen saturation (saturation < 92% during sedation), interventions such as verbal and physical contact, increase in oxygen flow, performing the planned painful procedure, basic airway maneuvers, insertion of naso‐ or oropharyngeal airway, bag‐mask ventilation, and administration of an antidote could be performed. Additionally, support from staff in the ED or the Department of Anesthesiology could be requested, typically in that order, but always at the discretion of the RM provider.

#### 2.5.3 Postprocedural Assessments

Providers monitored patients until fully awake and at least 10 min after the end of the procedure. When awake, patients were asked about procedural pain, satisfaction with sedation, and procedural recollection.

### 2.6 Data Analyses

To analyze for normal distribution, a QQ‐plot and Shapiro–Wilks tests were performed. Because all variables were nonparametric, descriptive statistics were presented as medians and interquartile ranges (IQR) (25th–75th percentile). Kruskal–Wallis tests were used to compare ASA I, ASA II, and ASA III, followed by Wilcoxon post hoc tests to identify pairwise differences. Chi‐square‐test or Fisher's exact tests were applied to compare proportions; Fisher's exact test was applied when any expected cell count was below 5. The Bonferroni correction was applied when appropriate. REDCap Research Electronic Data Capture tools were utilized to collect and manage all data (version 15.5.15, Vanderbilt University) [15, 16]. Data management and statistical analyses were done in SAS OnDemand for Academics (Version 9.4, SAS Institute Inc., Cary, NC, USA).

## 3 Results

In this study, 540 patients were included, and descriptive data are presented in Table 1. Gender distribution varied depending on ASA score, with ASA I having the minority of female patients and ASA III having the majority. As expected, ASA I patients were the youngest group.

**TABLE 1:** Demographic data, respiratory complications, and ASA score.

	ASA I (*n* = 113)	ASA II (*n* = 277)	ASA III (*n* = 149)	⋄
Age, median (IQR)	31 (22:58)	60 (42:76)	74 (64:86)	€$#
BMI, mg/cm^2^, median (IQR)	23.9 (21.4:26.35)	27.45 (24.5:31.05)	25.95 (23.4:30.75)	€#
Gender: women/men (%)	34.5%/65.5%	59.6%/40.4%	65.8%/34.2%	*
Potential airway risks: %, *n*				
None expected:	87.6%, 99	59.9%, 166	52.3%, 78	*
Risk factors present*	12.4%, 14	40.0%, 111	47.7%, 71	
*Large tongue (17.6%), decreased movement of neck (4.6%) or jaw (3.5%), obesity (17.4%), history of sleep apnoea (5.6%)				
Respiratory complications: %, *n*				
No or Mild/Moderate respiratory complications:	100%, 113	95.3%, 264	98.7%,147	*
No respiratory complications:	91.2%, 103	79.4%, 220	87.9%, 131	*
Mild/Moderate respiratory complications:	8.9%, 10	15.9%, 44	10.7%, 16	
Spoke loudly to the patient	7.1%, 8	12.3%, 34	5.4%, 8	*
Gently shook the patient	2.7%, 3	11.9%, 33	5.4%, 8	*
Increased oxygen	3.5%, 4	18.8%, 52	9.9%, 15	*
Started the procedure	0.9%, 1	10.5%, 29	6.0%, 9	*
Jaw thrust or oropharyngeal airway	1.8%, 2	17.0%, 47	6.7%, 10	
Severe respiratory complications:	0.0%, 0	4.7%, 13	1.3%, 2	*
Antidote usage	0.0%, 0	0.7%, 2	0.0%, 0	
Manual assisted ventilation	0.0%, 0	4.0%, 11	1.3%, 2	
Procedures: %, *n*				
Reduction procedure of hip joint	11.5%, 13	32.5%, 90	36.9%, 55	*
Reduction procedure of other joint	46.9%, 53	26.7%, 74	13.4%, 20	*
Lumbar puncture	4.4%, 5	3.6%, 10	4.7%, 7	
Fracture manipulation	31.9%, 36	24.9%, 69	23.5%, 35	
Painful movements	0.9%, 1	0.7%, 2	1.3%, 2	
Anxiety during procedure	0.9%, 1	0.7%, 2	2.7%, 4	
Other procedure, e.g., diagnostic imaging	3.5%, 4	10.1%, 28	17.5%, 26	*
Hernia reduction	0%, 0	0.7%, 2	0%, 0	

*Note:* ^⋄^One patient missing, ^€^Signifies a *p*‐value < 0.017 in comparison between groups ASA1/ASA2, ^$^between ASA2/ASA3, ^#^between ASA1/ASA3. *Signifies a *p*‐value < 0.017 in comparison between groups.

Abbreviations: ASA, American Society of Anesthesiologists; IQR, interquartile range; *n*, number.

### 3.1 RM Was Safe for ASA I–III Patients

In the present study, 95%–100% of patients in ASA I–III experienced no or mild/moderate respiratory complications, indicating overall safety across all groups, with the lowest complication rates observed in ASA I and III patients (Table 1, *p* = 0.014). ASA II patients had the most severe respiratory complications (*n* = 13), even though ASA III patients were the most vulnerable in terms of potential airway risks and comorbidity. Procedure types were similarly distributed across the ASA groups, although a trend was observed toward ASA II and III patients more often presenting with hip dislocations, whereas ASA I patients most frequently presented with fractures fitting the younger median age (Table 1).

### 3.2 RM Dose Was Age‐, Gender‐ and ASA‐Dependent

In this study, the RM dose was dependent on age, with younger patients needing higher doses (Table 2). Weight did not influence the required RM dose for effective sedation. When considering gender, men required the highest doses (*p* < 0.001), while ASA III patients required the lowest doses (*p* = 0.015). Opioid doses were lower in the higher ASA groups, reflecting the needs of more vulnerable and elderly patients (Table 2).

**TABLE 2:** Remimazolam doses and cotreatment with opioids.

Remimazolam dose	1.5 mg–< 10 mg (*n* = 195)	10 mg–< 20 mg (*n* = 258)	20 mg–< 30 mg (*n* = 66)	> 30 mg (*n* = 21)	
Age, median (IQR)	70 (47:80)	61 (42:74)	46 (29:67)	35 (22:61)	$§#€&
BMI, mg/cm^2^. median (IQR)	25.3 (22.7:28.9)	26.9 (23.8:30.3)	26.1 (24.6:33)	26.1 (22.6:30)	$§
Gender: women/men (%)	64%/36%	57%/43%	42%/58%	14%/86%	*
ASA score: %, *n*					
ASA I	19.0%, 37	20.5%, 53	21.2%, 14	42.9%, 9	*
ASA II	45.6%, 89	56.6%, 146	56.1%, 37	23.8%, 5	
ASA III	35.4%, 69	22.5%, 58	22.7%, 15	33.3%, 7	
**Opioids given within 60 min of the remimazolam administration**					
In total regardless of opioid type, %, *n* ^@^	38.5%, 75/195	43.0%, 111/258	36.0%, 24/66	28.6%, 6/21	
IV Morphine					
None %, *n*	84.1%, 164/195	78.7%,203/258	87.9%, 58/66	90.5%, 19/21	
Administered %, *n*	15.9%, 31/195	21.3%, 55/258	12.1%, 8/66	9.5%, 2/21	
Median dose (IQR)	5.0 (5.0;5.0)	5.0 (5.0;5.0)	5.0 (5.0;5.0)	3.8 (2.5;5.0)	
IV Fentanyl					
None %, *n*	78.0%, 152/195	78.3%, 202/258	75.8%, 50/66	81.0% 17/21	
Administered %, *n*	22.1%, 43/195	21.7%, 56/258	24.2%, 16/66	19.1% 4/21	
Median dose (IQR)	150 (100:250)	100 (100:200)	100 (100:250)	200 (100:350)	
IV Alfentanil					
None %, *n*	98.5%, 192/195	98.1%, 253/258	98.5%, 65/66	100%, 21/21	
Administered %, *n*	1.5%, 3/195	2.0%, 5/258	1.5%, 1/66	0.0%, 0/21	
**Opioids given during procedure**					
In total regardless of opioid type, %, n	71.3%, 139/195	84.2% 217/258	81.8%, 54/66	81.0%, 17/21	*
IV Fentanyl total dose					
None %, *n*	89.7%, 175/195	92.7% 239/258	92.4% 61/66	95.2%, 20/21	
Administered %, *n*	10.3%, 20/195	7.4%, 19/258	7.6%, 5/66	4.8%, 1/21	
IV Alfentanil total dose					
None %, *n*	33.9%, 66/195	17.5%, 45/258	18.2%, 12/66	19.1%, 4/21	*
Administered %, *n*	66.2%, 129/195	82.2%, 212/258	81.8%, 54/66	81.0%, 17/21	
Median dose (IQR)	0.5 (0.3:0.8)	0.8 (0.5:1.0)	0.9 (0.5:1.0)	0.8 (0.5:1.0)	

*Note:* *Signifies a *p*‐value < 0.013 in comparison between groups: $ = 1.5–< 10 vs. 10–< 20, § = 1.5–< 10 vs. 20–< 30, # = 1.5–10 vs. 30+, € = 10–20 vs. 20–30, & = 10–20 vs. 30+, £ = 20–30 vs. 30+. ^@^One patient had received IN Sufentanil, two patients had received PO Oxycodone, and one patient had received PO Tramadol.

Abbreviations: ASA, American Society of Anesthesiologists; IQR, interquartile range; *n*, number.

## 4 Discussion

In this quality study, 540 patients underwent procedural sedation with RM in the ED. The results demonstrated that RM had an equally favorable safety profile across ASA I–III patients, with ASA III patients experiencing fewer serious respiratory complications during procedural sedation than ASA II patients.

### 4.1 Safety

RM has demonstrated superior properties to the commonly used agents for ambulatory sedation, such as no injection pain, a higher success rate, a faster recovery time, and earlier patient discharge following procedures [4, 5]. The results of this study align with the study of Rex et al., where procedural sedation of ASA III/IV patients exhibited a safety profile comparable to that observed in lower‐risk ASA groups [9]. Our findings indicate that ASA I and III patients had a similar safety profile and presented with fewer and/or milder respiratory complications than ASA II patients, despite ASA III patients having a higher risk profile. The findings of this study align with the study of Pambianco et al., where RM was found safe to use on ASA III patients, with airway risk factors similarly distributed across groups. Additionally, similar safety profiles were observed between high‐risk and low‐risk ASA patients. However, in the study by Pambianco et al. ASA III patients with sleep apnea or obesity were excluded, due to an estimated higher risk of difficult airway management [17]. In this present study, however, patients with potential airway risks, including sleep apnea or obesity, were included if the one performing the RM sedation evaluated the patient as safe to receive the drug. This suggested that sleep apnea and obesity might not affect safety during procedural sedation with RM.

### 4.2 Doses

In the present study, we found, as expected, that the RM dose required for sedation was consistent with the findings of Eleveld et al. and was not influenced by patient weight, consistent with the known pharmacology of benzodiazepines [18, 19]. Men tended to receive higher doses than women, and ASA III patients typically received lower doses than ASA I and II, consistent with expectations due to their older age and increased frailty. The general RM doses administered in this study were higher than those recommended in local guidelines; however, this did not impact the safety, with no severe adverse events occurring among patients receiving doses above the recommended limit. Notably, two ASA III patients did experience severe respiratory complications, but both were administered doses lower than the 17.5 mg recommended in local guidelines [20].

### 4.3 Preventive Properties

RM shows potential for reducing time spent in hospital in comparison to propofol [5]. This approach may be more cost‐effective, and a study by Pedersen et al. found procedural sedation with RM economically beneficial in terms of reduced length of hospital stays compared to procedural sedation with midazolam and propofol in colonoscopies and bronchoscopies [21].

In future studies on procedural sedation with RM, it would be highly relevant to investigate the health economic consequences associated with the procedure. Additionally, it would be relevant to research whether procedural sedation with RM routinely requires the addition of an opioid, since almost all patients in this study were administered some form of opioid in addition to the RM.

### 4.4 Strengths and Limitations

This study included patients dependent on provider availability during shifts rather than following a predefined inclusion protocol. Patients were selected opportunistically and not through a randomized clinical trial (RCT), making it susceptible to selection bias. Therefore, an RCT study would be a stronger approach. However, this study had clear inclusion and exclusion protocols, strengthening the internal validity. Due to study design, excluded patients were not registered and data regarding those are therefore not available. Since this study had a large study population, the evidence of our findings is strengthened. The diverse patient population and broad clinical relevance means that our results can be used in other clinical settings, increasing the external validity. Due to the large group of providers, there may have been variability in the assessment of patient condition, which introduces information bias. This study did not inquire about patients' previous drug or substance use, which could explain the higher doses of RM administered.

## 5 Conclusion

This study demonstrated that RM has a promising potential for procedural sedation with a high safety profile for ASA I–III patients even with high doses and in high‐risk profiles. RM appears to be a viable option for a variety of procedures in the ED, and procedural sedation of ASA III patients with RM was safe regardless of potential airway risks. The RM dose was found to be age, gender and ASA score dependent. However, further randomized multicenter studies are required to confirm the findings of this study and enhance the generalizability and applicability in broader clinical settings.

## Author Contributions

C.H.B. contributed with conceptualization, analysis and interpretation of data, manuscript drafting, software and statistical analysis, review and editing. S.P.B. contributed with conceptualization, analysis and interpretation of data, manuscript drafting, software and statistical analysis, review and editing. A.L.K. contributed with conceptualization, analysis and interpretation of data, software and statistical analysis, critical revision of the manuscript for important intellectual content, statistical expertise, acquisition of data, resources, supervision, and project administration. D.M. contributed with conceptualization, critical revision of the manuscript for important intellectual content, resources, supervision, and project administration.

## Funding

The authors have nothing to report.

## Conflicts of Interest

The authors declare no conflicts of interest.

## Data Availability

The data that support the findings of this study are available from the corresponding author upon reasonable request.
